# Prognosticating for Adult Patients With Advanced Incurable Cancer: a Needed Oncologist Skill

**DOI:** 10.1007/s11864-019-0698-2

**Published:** 2020-01-16

**Authors:** Christina Chu, Rebecca Anderson, Nicola White, Patrick Stone

**Affiliations:** Marie Curie Palliative Care Research Department, Division of Psychiatry, University College London (UCL), 6th Floor, Maple House, 149 Tottenham Court Road, London, W1T 7NF UK

**Keywords:** Prognosis, Prediction, Palliative care, Neoplasms, Communication, Uncertainty

## Abstract

Patients with advanced cancer and their families commonly seek information about prognosis to aid decision-making in medical (e.g. surrounding treatment), psychological (e.g. saying goodbye), and social (e.g. getting affairs in order) domains. Oncologists therefore have a responsibility to identify and address these requests by formulating and sensitively communicating information about prognosis. Current evidence suggests that clinician predictions are correlated with actual survival but tend to be overestimations. In an attempt to cultivate prognostic skills, it is recommended that clinicians practice formulating and recording subjective estimates of prognosis in advanced cancer patient’s medical notes. When possible, a multi-professional prognostic estimate should be sought as these may be more accurate than individual predictions alone. Clinicians may consider auditing the accuracy of their predictions periodically and using feedback from this process to improve their prognostic skills.

Clinicians may also consider using validated prognostic tools to complement their clinical judgements. However, there is currently only limited evidence about the comparative accuracy of different prognostic tools or the extent to which these measures are superior to clinical judgement. Oncologists and palliative care physicians should ensure that they receive adequate training in advanced communication skills, which builds upon their pre-existing skills, to sensitively deliver information on prognosis. In particular, clinicians should acknowledge their own prognostic uncertainty and should emphasise the supportive care that can continue to be provided after stopping cancer-directed therapies.

## Introduction

Prognostication is the process of making predictions about future health outcomes, usually about predicting length of survival. Patients with advanced cancer frequently express a desire for prognostic information [1]. However, simply being told that one is “terminal” or “incurable” does not usually provide sufficient information for patients or families to make detailed plans for the future [1, 2]. Even if patients do not wish to know exact timescales, they may want to have prognostic information to inform treatment discussions, undertake advance care planning, or help with decision-making [3, 4]. Clinicians commonly find it difficult to make these predictions and to discuss them with patients and relatives, leading to unmet information needs [5, 6]. Communication in this area is complex and requires skill and experience, particularly in finding the balance between conveying useful information about expected timeframes, while also explaining the inherent uncertainty in such predictions. Advanced communication skills should therefore be considered an essential element in the process of prognostication [7].

The prognosis of an individual patient is liable to change over time and to be influenced by biological, clinical, and social factors beyond diagnosis and stage of disease [8, 9]. Therefore, it is important for oncologists to develop and maintain skills in predicting survival even when the disease has become incurable and disease-directed treatment options are limited or non-existent. This is distinct from the more common practice of staging cancers to derive median survival estimates at diagnosis or the use of prognostic markers to predict response to treatment.

A prognostic estimate can be formulated using a clinician’s experience and knowledge to make a judgement; this is known as a clinical prediction of survival. Although widely used, there are concerns about the subjective nature of this approach, which makes it difficult to reproduce and teach. Also, subjective judgements tend to be inaccurate and over-optimistic [10, 11^••^, 12]. Therefore, more “objective” scales and tools have been developed to support clinical predictions [13, 14^••^].

The aim of this paper is to evaluate the recent literature on prognostication for patients with advanced cancer and to suggest ways in which physicians can improve their own clinical practice in formulating and communicating prognostic estimates.

## Clinical prognostic estimates

Clinical prognostic estimates usually take one of two formats: temporal (how long?) or probabilistic (how likely?). Temporal predictions can either be specific (e.g. “two days”) or can use broader categories (e.g. “one to two months”). Probabilistic predictions estimate the likelihood of patients surviving to specific time points (e.g. “70% chance of surviving two weeks”). An alternative option is for clinicians to ask themselves the Surprise Question (SQ); “would I be surprised if this patient were to die in the next 6-12 months?” [15]. The SQ was developed to prompt clinicians to consider whether patients would benefit from a shift in focus of care or from referral to specialist palliative care services, rather than as a method of prognostication per se. The original SQ has been modified to use different time frames (e.g. 30 days) [16, 17], or to consider survival rather than death; “would I be surprised if this patient were to be alive in the next 6-12 months?” [18, 19]. The SQ is a prompt for introspection, encouraging clinicians to consider the possibility that the patient may die in the foreseeable future rather than asking them to make a specific prediction about how long they will live.

### How accurate are clinical predictions of survival?

#### Temporal and probabilistic predictions

There is a growing body of evidence about the accuracy of clinician predictions of survival. In preparation for this review, the authors identified two relevant systematic reviews [10, 11^••^] and ten further articles published in the last 4 years (summarised in Table 1) [20–29].

**Table 1:** Summary of recent studies of the accuracy of clinician predictions of survival (June 2015 to June 2019)

	Setting	Patients	Prediction	Accuracy defined as	Outcome (95% CI, if available)
Temporal predictions					
Amano 2016	16 palliative care units, 19 hospital palliative care teams, 23 home palliative care services Japan	*N* = 2036 Locally advanced or metastatic cancer	Specific temporal (for additional analysis also separated into categories < 7 days, 7–13 days, 14–27 days, 28–41 days, 42–55 days, 56–83 days, and 84 days+)	CPS lies between 0.67 and 1.33* times the AS, i.e. error was less than + 33% Spearman’s correlation coefficient between CPS and AS	- Accurate in 35% (33–37%); overestimated in 45% (43–47%) - Categories concordant 35% - Spearman’s correlation coefficient 0.69 reflecting good agreement
Hui 2016 and Farinholt 2018	Hospital, USA; secondary analysis of study of new prognostic marker	*N* = 222 Advanced cancer with palliative care consults	Specific temporal	C-index. AUC at 30 days and 100 days	- C-index 0.58 (0.47–0.68) - AUC at 30 days 0.58 (0.47–0.68) - AUC at 100 days 0.62 (0.54–0.70)
Urahama 2018^	Hospice, Japan	*N* = 101 Cancer referred from hospital to hospice for end of life care	Specific temporal	CPS lies between 0.67 and 1.33* times the AS, i.e. error was less than + 33%	- Accurate in 22.8%; overestimated in 67.3%
Tavares 2018	Hospital, Portugal	*N* = 38 Cancer known to the palliative care team (excluded haematological malignancy)	Specific temporal	If CPS and actual survival differed by less than 1 week	- Junior doctors accurate in 10.5% - Palliative care physicians accurate in 23.7%
Razvi 2018	Hospital, Canada	*N* = 172 Advanced cancer referred for palliative radiotherapy	Specific temporal (for additional analysis converted to categories ≤12, 13–26, 27–52, > 52 weeks)	Mean and median difference between CPS and AS in weeks	- Median difference overestimated by 14 weeks (IQ 1.3–36.4) - Categories concordant 41%
Vasista 2019	Secondary analysis of INTEGRATE trial of regorafenib in multiple countries (Australia, New Zealand, Canada, South Korea)	*N* = 152 Locally recurrent or metastatic gastric or oesophago-gastric cancer progressing after 1 or 2 lines of chemotherapy, ECOG status 0–1	Specific temporal	CPS lies between 0.67 and 1.33* times the AS, i.e. error was less than + 33% C statistic	- Accurate in 29% - C-index 0.62 (0.57–0.68)
Gramling 2019	2 hospitals, USA	*N* = 230 Metastatic cancer referred for palliative care consultation with a clear comfort only plan (excluded haematological malignancy)	Categorical temporal (< 24 h, 24 h to < 2 weeks, 2 weeks to < 3 months, 3 months to < 6 months, and > 6 months)	If category selected by physician was concordant with actual survival	- Accurate in 41%, overestimate in 50%^#^
Simmons 2019	9 regional cancer centres, 7 palliative care units, UK	*N* = 463 Incurable cancer (excluded breast or prostate cancer with only bone metastasis)	Categorical temporal (≤14 days, 15–56 days, and ≥ 57 days)	AUC at 1 and 3 months	- AUC 1 month 0.71 (0.63–0.79) - AUC at 3 months 0.68 (0.62–0.73)
Ermacora 2019	2 palliative care units, Italy	*N* = 334 Advanced cancer with no indications for oncology treatments, KPS 10–50%	Categorical temporal (1–2, 3–4, 5–6, 7–10, 11–12 weeks, or over 12 weeks)	AUC at 30 days separated by who made the prediction	- Nurse AUC 0.78 (0.72–0.82) - Physician 1 AUC 0.77 (0.77–0.81) - Physician 2 AUC 0.76 (0.71–0.81)
Probabilistic predictions					
Malhotra 2019	Outpatient oncology, USA; secondary analysis of VOICES trial (communication and decision-making intervention)	*N* = 263 Stage IV non-haematological cancer or stage III plus oncologist would “not be surprised” if the patient died within 12 months	Probabilistic—chances of survival at 2 years with options of 0%, about 10%, about 25%, about 50%, about 75%, about 90%, or 100%	If difference between status at 2 years and prediction − 0.49 to 0.49^+^ AUC at 2 years	- Accurate predictions 62% - Pessimistic predictions 26% - AUC 0.81 (0.75–0.87)

*CPS* clinician predicted survival, *AS* actual survival, *AUC* area under the curve (measure of concordance commonly used to evaluate predictive ability of a model; general agreement a value > 0.7 is acceptable)

*CPS/AS

^Results only from oncologist estimates; the palliative physicians are suggested to use predictive models to prognosticate

^#^Extrapolated from data provided in paper

^+^Status at 2 years (alive = 1 and dead = 0) minus prediction (i.e. 0, 0.1, 0.25, 0.5, 0.72, 0.9, or 1)

Clinician predictions are correlated with actual survival, but there is a wide variation in reported accuracy, with a general tendency to overestimate [10, 11^••^, 12, 20–23]. Clinicians’ specific predictions range from underestimating by half to a sixfold overestimation [10, 11^••^]. One large prospective cohort study of 2036 locally advanced or metastatic cancer patients accessing palliative care services found that 45% predictions were overestimates, 35% accurate, and 20% underestimates [20].

There is a lack of consensus in the literature about what constitutes an “accurate” prediction, which makes interpretation of study results challenging. For example, should a prediction of 12 weeks be considered “accurate” if the patient dies 2 weeks before or after this? For specific temporal predictions, a common approach is to regard a prognosis as accurate if actual survival falls within ± 33% of the prediction [20, 21, 25, 30]. One limitation of this definition is that the magnitude of the absolute permissible error is small when the estimated prognosis is short and is fairly large when the estimated prognosis is longer. Thus, a clinical prediction of survival of 3 days may be judged inaccurate if the patient dies in less than 48 h, whereas a prediction of 3 months may be considered accurate even if the patient dies 4 weeks earlier than expected. The level of “inaccuracy” that is clinically significant remains unclear; at what point does a more “accurate” prognosis alter the management plan or come to be considered important by patients or their relatives?

The issue of defining “accuracy” is magnified for probabilistic estimates. How, for instance, should one judge the accuracy of a prediction that a patient has 40% chance of surviving 2 weeks? A similar conundrum confronts weather forecasters who often provide probabilistic estimates about, for example, the chances of rain tomorrow. Professional forecasters tend to judge probabilistic predictions using the Brier score [31]. This statistic reflects the degree to which a prediction was correct or incorrect. Thus, a prediction of 90% is “more correct” than a prediction of 70% if the event did actually occur. Brier scores range between 0 (accurate) and 1 (inaccurate) with a score of 0.25 representing a prediction of 50%. To our knowledge, only two studies have utilised the Brier score to assess the accuracy of clinicians’ survival predictions [32, 33^•^]. The mean Brier scores in these studies were 0.22 and 0.24 suggesting clinician’s probabilistic predictions are slightly better than chance.

There is mixed evidence about whether certain health professionals are better prognosticators than others or whether prognostic ability improves with experience or seniority [11^••^]. However, there is some evidence that multi-professional estimates are more accurate than predictions made by individual clinicians alone [34, 35]. In order to develop expertise, repeated “deliberate practice” is required [36, 37]. Deliberate practice is a structured activity that explicitly aims to improve performance by reflecting on the results of the performance and gaining feedback. Therefore, one might suppose that clinicians ought to be able to improve the accuracy of their prognoses through deliberate practice, although this hypothesis has not yet been tested.

In summary, despite the significant methodological difficulties associated with judging prognostic accuracy, there is fairly consistent evidence that clinicians’ estimates correlate with actual survival. It appears that clinicians, regardless of the method of prognostication, do recognise when patients are deteriorating.

#### Surprise Question

In systematic reviews, the SQ tends to be more accurate in cancer patients than in patients with other life-threatening conditions [38, 39]. For patients with cancer, the SQ has a positive predictive value (proportion of patients who die when the clinician would not be surprised by their death) of 46.8–49.3%, a negative predictive value (proportion of patients who survive when the clinician would be surprised by their death) of 92.3–92.4%, a sensitivity (the proportion of those correctly identified as dying) of 77.1%, and a specificity (proportion of patients correctly identified as surviving) of 84.3%. Referral to palliative care services is seldom based solely on having a poor prognosis; however, early identification of palliative care patients is often triggered by a combination of an expected prognosis of less than a year, combined with complex needs and/or patient choice. Based on the psychometric properties of the SQ, its greatest clinical value may lie in its negative predictive value for “screening out” patients who are unlikely to need immediate referral to specialist palliative care services (all other things being equal). It is, however, important to remember that patients’ conditions change and that their prognosis is therefore in need of regular re-appraisal.

### Predicting imminent death

#### Are clinicians more accurate at predicting imminent death?

The concept of the “horizon effect” suggests that predictions ought to become more accurate as the event being predicted draws closer [8]. There is conflicting evidence in the literature about whether this is true for survival predictions [20, 22, 23, 30, 40–44]. In one study, clinicians were asked to provide repeated specific temporal and probabilistic predictions for the following 24 and 48 h, for patients admitted to a palliative care unit [30]. Temporal predictions were defined as accurate if there was an error of less than ± 33%. Probabilistic predictions were considered accurate if the clinician predicted ≤ 30% survival and the patient died, or if the clinician predicted ≥ 70% survival and the patient survived. Using these definitions, the authors concluded that probabilistic estimates were more accurate than temporal predictions throughout the study period. However, the accuracy of temporal predictions remained constant, whereas the accuracy of probabilistic estimates worsened over the last 14 days of life. It is important to note that there have only been a limited number of studies specifically investigating imminent death and the majority have been conducted with patients who have already been identified as suitable for palliative care referral or admission to a palliative care unit. Therefore, these results may not hold true for unselected patient groups on general oncology wards, especially those admitted with acute illnesses.

#### How do clinicians recognise the last days of life?

In a Delphi survey of international palliative care experts, there was over 50% consensus that general deterioration in physical condition, reduced levels of consciousness and cognition, decreased intake of fluid and food, skin changes, altered emotional state, and specific patterns of breathing were features that predicted when patients were entering the last hours and days of life [45]. However, many clinicians also feel they can intuitively recognise when a patient is imminently dying. In semi-structured interviews, staff working in oncology and cardiology wards were asked how they recognised patients who were dying. A range of symptoms, signs, imaging, and laboratory results were reported; however, participants found it very difficult to describe how they assimilated this information, commonly using terms such as having a “subconscious” feeling or a “sixth sense’” that someone was about to die [46].

One way to better understand this so-called sixth sense is to use a research method known as Judgement Analysis [47]. White and colleagues used this approach to investigate which factors subconsciously influenced clinicians’ opinions about whether a patient was about to die within the next 72 h [33^•^]. First, expert prognosticators were selected by their superior ability to predict imminent death using clinical vignettes based on real patient cases; the expert group had a mean Brier score of 0.184, reflecting better predictive accuracy, compared to the whole group mean of 0.237. Next, by altering clinical parameters in fictional cases, the extent to which certain factors affected the experts’ judgements could be established. The most influential factor in determining whether clinicians thought that patients were about to imminently die was the Palliative Performance Scale (PPS), followed by the presence or absence of Cheyne-Stokes breathing, general deterioration in the patient’s condition, and level of sedation or agitation. Knowing what factors expert prognosticators subconsciously use to formulate their predictions could potentially allow others to learn how to improve their estimates and to better recognise patients who are imminently dying. A randomised controlled trial demonstrates that the use of an online training programme can teach medical students to adopt similar judgement strategies to experts [48]. However, further work will be required to determine how the learning can be consolidated and whether the skills are transferable from the classroom to clinic.

Another method to understand how clinicians might recognise patients who are imminently dying is to identify signs and symptoms that may be pathognomonic of the dying process [49]. In a palliative care context, vital signs (e.g. pulse, blood pressure, and oxygen saturations) have a low sensitivity or positive predictive value for identifying those who are imminently dying, supporting the practice of not routinely undertaking these observations in terminal care settings [50]. However, Hui and colleagues have identified 13 clinical signs which have high specificity (> 95%) and positive likelihood ratios (> 5) of predicting the final 72 h of life [51, 52]. Sensitivity of these signs rarely reached over 30%, meaning their absence does not exclude imminent death, limiting their use in clinical practice. Nonetheless, this work has informed the development of a clinical tool to aid the diagnosis of impending death [53]. The initial model combines the PPS as a tool to “rule out”, and “drooping of nasolabial folds” as a specific sign to help “rule in”, impending death. A second model utilises the other 12 signs identified in these studies. Further development and validation is required before this approach can be recommended for clinical use.

## Performance scales and prognostic tools

Performance or functional scales provide a systematic approach to evaluate general well-being and ability to perform activities of daily living. They require clinicians to match signs and symptoms of patients to a description of function on a scale. The Karnofsky Performance Status (KPS) was the first to be developed and consists of single statements describing levels of function from 0 to 100% [54]; the Australian Modified KPS (AKPS) involves a slight alteration to the phrasing of the statements to provide greater discrimination between certain levels [55]. The PPS is also a modification of the Karnofsky, in which there are five descriptive domains of function rather than a single descriptor [56]. In contrast, the Eastern Cooperative Oncology Group (ECOG) performance scale consists of only five levels [57]. Although not specifically devised as prognostic tools, performance scales have been shown to correlate with survival in a variety of settings [58–60].

In addition to performance scales, there are several tools that have been specifically developed with the aim to predict survival in patients with advanced cancer. A recent systematic review identified seven suitably validated prognostic tools [14^••^]: PPS [56], Palliative Prognostic Score (PaP) [61], Delirium Palliative Prognostic Score (D-PaP) [62], Palliative Prognostic Index (PPI) [63], Glasgow Prognostic Score (GPS) [64], B12/CRP index (BCI) [65], and the Prognosis in Palliative Care study predictor models (PiPS-A and PiPS-B) [34]. A summary of these tools is provided in Table 2.

**Table 2:** Summary of performance scales and prognostic tools validated in advanced cancer populations

Prognostic scale or tool	Prediction made	Factors included in the scale or tool	Comments
Palliative Performance Scale (PPS)	Patient assigned to a decile (from 100 to 0%). Each decile shows a distinct ordered survival difference with lower deciles associated with shorter survival.	Users find the “best fit” across the following domains to assign a decile •Ambulation •Activity and evidence of disease •Self-care •Intake (food and fluid) •Conscious level	- Does not rely on blood results or clinician predictions of survival. - Greatest accuracy found at the lower levels; a PPS of 10%, 20%, and 30% had a median survival of 2, 4, and 13 days respectively.
Palliative Prognostic Score (PaP)	Cumulative total assigns patients to one of three groups with < 30%, 30–70%, or > 70% probability of surviving 30 days.	Each component assigns a score towards a cumulative total •Symptoms of dyspnoea •Symptoms of anorexia •Karnofsky performance status •Clinician predicted survival •White cell count •Lymphocyte %	- Clinician predicted survival is weighted heavily within the scoring system - Requires blood tests to be taken
Delirium Palliative Prognostic Score (D-PaP)		Same factors as PaP with the addition of presence or absence of delirium	
Palliative Prognostic Index (PPI)	Score assigns patients to one of three groups of estimated survival of < 3 weeks, 3–6 weeks, or < 6 weeks	Each component assigns a score towards a cumulative total •Palliative performance scale •Oral intake •Presence of oedema •Presence of delirium •Symptoms of dyspnoea	- Does not rely on blood results or clinician predictions of survival - Contains PPS within the scoring system; it is unclear whether the PPI provides additional prognostic accuracy above PPS alone
GPS (Glasgow Prognostic Score) or mGPS (modified GPS)	Score assigns patients to one of three groups: good, intermediate, and poor prognostic groups	Each component assigns a score towards a cumulative total •C-reactive protein •Albumin	- Requires blood tests to be taken - Allocation to prognostic group does not give indication of survival time; individual studies have to be found to understand median survival times for specific cancer groups, e.g. gastric cancer
B12/CRP Index (BCI)	Score assigns patients to one of three groups with % probability of surviving 90 days	Total score derived from multiplying •Serum vitamin B12 level (pmol/L) and •Serum CRP level (mg/L)	- Requires blood tests to be taken - The only confirmatory study conducted found partial support for the BCI; they did not find statistically significant differences between all prognostic groups
Prognosis in Palliative care study (PiPS - A) score	Provides a probability of surviving days (0–14 days), weeks (15–55 days), or months (> 55 days)	Prediction is based on a regression equation using the following variables •Diagnostic information •Sites of metastases •Presence or absence of key symptoms •Cognitive status •ECOG functional status	- Does not rely on blood results or clinician predictions of survival - Results can be calculated using an online calculator
Prognosis in Palliative care study (PiPS - B) score		Similar factors as for PiPS-A but with addition of blood results	- Does not rely on clinician predictions of survival. - In one study was found to be better than a doctor’s or a nurse’s survival prediction

The place of prognostic tools in clinical practice has not yet been clearly defined. Despite validation in relatively large numbers of advanced cancer patients, which has demonstrated adequate discrimination and calibration, there has been limited comparison of their performance against each other or against clinicians’ predictions. This means it is often unclear whether these tools perform better, worse, or the same as clinicians making predictions alone. Moreover, it is not always easy to judge how the output of these prognostic tools should be interpreted. The PaP, for instance, predicts whether patients will have a < 30%, a 30–70%, or a > 70% chance of surviving for the next 30 days. However, it is not clear how one should interpret these results on an individual patient basis. What does a 30–70% chance of surviving 30 days mean, and how should it be acted upon? Another problem that arises is knowing which tool to use at what time. For example, the PaP and PPI both provide results that may help to distinguish between whether patients will survive for weeks or months, but neither is designed to identify when patients are entering the final days of life. In addition, whether certain tools perform better for patients with certain types of cancers is unknown. These limitations mean that existing prognostic tools cannot yet be recommended as a replacement for clinicians’ predictions. For the moment, they should perhaps best be considered as a means of triangulating, or cross-checking, clinicians’ own clinical intuitions. They may also have a specific role when clinicians doubt their own judgement or when an “objective” estimate is required, for example for benchmarking of clinical services or determining inclusion and exclusion criteria for clinical trials.

## Communicating the prognostic estimate

### Initiating prognostic discussions

Clinicians often find it difficult to talk about prognosis. Research has shown clinicians sometimes “collude” with patients in creating false optimism, or miss or avoid cues from patients to talk about prognosis, moving straight to talking about treatment options [66, 67]. The desire to focus on the positives is understandable, particularly if it is not clear whether patients are ready to talk about their prognosis. Clinicians therefore need strategies for initiating prognostic conversations sensitively and to pick up on signs from patients that they may be ready to talk about these issues. A recent study recorded hospice outpatient consultations and showed that patients often displayed signs of wanting to discuss prognosis when given the opportunity to influence the consultation [68^••^, 69]. Patients frequently requested prognostic information by using statements (e.g. “I don’t know when it’s coming”) rather than asking direct questions. These statements allowed doctors to proceed to checking patients’ current understanding and their perspectives, and to re-confirm their readiness to hear a prognosis, before delivering it [68^••^].

### Content of prognostic discussions

When a patient’s condition first becomes incurable, information about how their function may deteriorate can be as important as life expectancy estimates in allowing patients to pursue achievable goals and make practical plans for future decline [70]. This information could also help inform their decisions about treatment, as more aggressive care could reduce their ability to “live well” until they die [71]. Over time, patients’ goals and prospects are likely to change and so a step-wise approach of continued conversations about prognosis is needed [72]. When patients reach the end of cancer-directed therapies, clinicians can focus on the care that will continue to be provided rather than describing palliative and supportive care as “doing nothing” [73, 74]. Prognostic discussions are an opportunity to reframe hope by being honest about the difficulties the patient is facing and emphasising symptom control, continuing relationships between clinicians and patients, and establishing achievable goals that optimise quality of life [75]. As the disease progresses, function becomes less relevant and time is more important to allow patients and relatives to make necessary arrangements, say goodbye, and provide the opportunity for relatives to be present when the patient dies [76]. Research has also shown that families often have a greater desire for prognostic information than patients when death is imminent [77, 78]. It is therefore important to take the patient’s lead on how much information to provide and be aware that separate conversations with families may be needed [79].

Prognostic uncertainty and lack of confidence discussing this with patients are key reasons for clinicians to avoid prognostic conversations, and so learning how to communicate an uncertain prognosis is important [80]. Current evidence-based guidelines emphasise the importance of stating prognostic uncertainty, but this is not the same as saying, “I do not know” or “it’s impossible to judge” [81–83, 84^••^]. There is no evidence to suggest whether providing estimates in temporal or probabilistic terms is the better approach when communicating prognosis. However, the use of numerical information in doctor-patient communication has been found to be contentious and the way in which it is received shifts depending on context and stage of illness [85]. In this study, patients felt statistics were used by clinicians with inadequate communication skills to subvert the need to confront difficult emotions or discussions. In practice, clinicians commonly use broad categories (e.g. days to weeks) to discuss prognosis with advanced cancer patients [86]. This approach is a useful way to couch prognostic uncertainty, as it provides patients and families with a meaningful prognosis without giving a spuriously precise estimate [87].

### Building skills to improve prognostic discussions

Recent trials of communication training for oncologists have shown some promising results. Interventions have shown improvement in patient-centred and empathetic communication, earlier and more regular prognostic conversations, and reductions in anxiety, but have struggled to improve prognostic awareness and goal-concordant care [88, 89, 90^•^, 91]. Examination of real-life conversations may help identify which elements of communication are most able to influence the interaction. The “Real Talk” training programme provides learning points based on analysis of naturally occurring conversations and uses clips to provide real-life examples for trainees [92]. A similar approach has been used in communication training in other settings with promising results [93, 94]. Oncologists and palliative care clinicians have more experience of discussing prognosis than most specialists, and so the approach of basing recommendations on real-life interactions, and building on clinicians’ pre-existing skills, could be a useful learning strategy for other less-experienced clinicians. Any guidance or training for oncologists about how to communicate prognosis must also provide them with skills to respond appropriately to expressions of emotion. Derry and colleagues suggest various strategies to address emotional responses such as allowing silence, validating emotions, and signposting to psychological services where necessary [95^•^]. There are also a number of organisational factors that could contribute to better communication of prognosis. These include allowing sufficient time to discuss prognosis, allowing patients to ask questions [96], better integration of oncology with palliative care [97], and support for clinicians through encouragement of reflective practice [98].

## Conclusion and recommendations

The ability to formulate and communicate a prognosis has advantages for the patient, their families, and for clinical teams. Viewing prognostication as a clinical skill invites us to understand that this is something that can be practised and improved. Taking note of one’s own prognostic predictions and following them up may help practitioners to practice reflectively and to hone their prognostic skills through feedback. When possible, a discussion with a multi-professional team should be conducted as this may help refine the prognostic estimate. Practitioners should also consider using prognostic tools to support their decision-making, but should remain aware that the prognostic tools themselves are also frequently inaccurate and should currently only be used as an aid towards, rather than a replacement of, clinical decision-making.

Clinicians find talking about prognosis difficult, both in terms of how to initiate these conversations and how to conduct them. Offering opportunities to ask questions or encouraging patients to elaborate on their thoughts allows them to steer the consultation, if they wish, towards prognostic discussions. If patients make statements that hint that they are ready to start having prognostic discussions, these should be explored, not ignored. Uncertainty is a key element that needs to be conveyed during these conversations. On a practical level, this can often be achieved by describing prognosis in terms of general time frames (e.g. “hours”, “days”, “weeks”, “months”, or “years”) rather than using precise estimates or complex statistical concepts such as probability or risk.

## References

[1] Innes S, Payne S (2009). Advanced cancer patients’ prognostic information preferences: a review. Palliat Med.

[2] Rohde G, Söderhamn U, Vistad I (2019). Reflections on communication of disease prognosis and life expectancy by patients with colorectal cancer undergoing palliative care: a qualitative study. BMJ Open.

[3] Steinhauser KE, Christakis NA, Clipp EC, McNeilly M, McIntyre L, Tulsky JA (2000). Factors considered important at the end of life by patients, family, physicians, and other care providers. JAMA..

[4] LeBlanc TW, Temel JS, Helft PR (2018). “How Much Time Do I Have?”: communicating prognosis in the era of exceptional responders. American Society of Clinical Oncology Educational Book.

[5] Hancock K, Clayton JM, Parker SM, Wal der S, Butow PN, Carrick S (2007). Truth-telling in discussing prognosis in advanced life-limiting illnesses: a systematic review. Palliat Med.

[6] Mack JW, Smith TJ (2012). Reasons why physicians do not have discussions about poor prognosis, why it matters, and what can be improved. J Clin Oncol.

[7] Christakis N. Death foretold: prophecy and prognosis in medical care. 1st ed. University of Chicago Press; 1999.

[8] Glare PA, Sinclair CT (2008). Palliative medicine review: prognostication. J Palliat Med.

[9] Croft P, Altman DG, Deeks JJ, Dunn KM, Hay AD, Hemingway H, LeResche L, Peat G, Perel P, Petersen SE, Riley RD, Roberts I, Sharpe M, Stevens RJ, van der Windt D, von Korff M, Timmis A (2015). The science of clinical practice: disease diagnosis or patient prognosis? Evidence about "what is likely to happen" should shape clinical practice. BMC Med.

[10] Cheon S, Agarwal A, Popovic M, Milakovic M, Lam M, Fu W (2015). The accuracy of clinicians’ predictions of survival in advanced cancer: a review. Annals of Palliative Medicine.

[CR11] White N, Reid F, Harris A, Harries P, Stone PA (2016). Systematic review of predictions of survival in palliative care: how accurate are clinicians and who are the experts?. PLOS ONE..

[12] Glare P, Virik K, Jones M, Hudson M, Eychmuller S, Simes J, Christakis N (2003). A systematic review of physicians’ survival predictions in terminally ill cancer patients. BMJ..

[13] Maltoni M, Caraceni A, Brunelli C, Broeckaert B, Christakis N, Eychmueller S, Glare P, Nabal M, Viganò A, Larkin P, de Conno F, Hanks G, Kaasa S, Steering Committee of the European Association for Palliative Care (2005). Prognostic factors in advanced cancer patients: evidence-based clinical recommendations--a study by the Steering Committee of the European Association for Palliative Care. J Clin Oncol.

[CR14] Simmons CPL, McMillan DC, McWilliams K, Sande TA, Fearon KC, Tuck S (2017). Prognostic tools in patients with advanced cancer: a systematic review. Journal of Pain and Symptom Management.

[15] Lynn J (2005). Living long in fragile health: the new demographics shape end of life care. Hastings Cent Rep.

[16] Cohen LM, Ruthazer R, Moss AH, Germain MJ (2010). Predicting six-month mortality for patients who are on maintenance hemodialysis. CJASN..

[17] Hamano J, Morita T, Inoue S, Ikenaga M, Matsumoto Y, Sekine R (2015). Surprise questions for survival prediction in patients with advanced cancer: a multicenter prospective cohort study. Oncologist..

[18] Weijers F, Veldhoven C, Verhagen C, Vissers K, Engels Y (2018). Adding a second surprise question triggers general practitioners to increase the thoroughness of palliative care planning: results of a pilot RCT with case vignettes. BMC Palliat Care..

[19] Royal College of General Practitioners. Matters of life and death: helping people to live well until they die. General practice guidance for implementing the RCGP/RCN end of life care patient charter; 2012.

[20] Amano K, Maeda I, Shimoyama S, Shinjo T, Shirayama H, Yamada T (2015). The accuracy of physicians’ clinical predictions of survival in patients with advanced cancer. Journal of Pain and Symptom Management.

[21] Urahama N, Sono J, Yoshinaga K (2018). Comparison of the accuracy and characteristics of the prognostic prediction of survival of identical terminally ill cancer patients by oncologists and palliative care physicians. Jpn J Clin Oncol.

[22] Razvi Y, Chan S, Zhang L, Tsao M, Barnes E, Danjoux C (2018). Are we better a decade later in the accuracy of survival prediction by palliative radiation oncologists?. Annals of Palliative Medicine.

[23] Gramling R, Gajary-Coots E, Cimino J, Fiscella K, Epstein R, Ladwig S, Anderson W, Alexander SC, Han PK, Gramling D, Norton SA (2019). Palliative care clinician overestimation of survival in advanced cancer: disparities and association with end-of-life care. J Pain Symptom Manag.

[24] Ermacora P, Mazzer M, Isola M, Pascoletti G, Gregoraci G, Basile D, de Carlo E, Merlo V, Luz O, Cattaruzza M, Orlando A, Bozza C, Pella N, Sacco CS, Puglisi F, Fasola G, Aprile G (2019). Prognostic evaluation in palliative care: final results from a prospective cohort study. Support Care Cancer.

[25] Vasista A, Stockler M, Martin A, Pavlakis N, Sjoquist K, Goldstein D et al. Accuracy and prognostic significance of oncologists’ estimates and scenarios for survival time in advanced gastric cancer. Oncologist. 2019. doi: 10.1634/theoncologist.2018-0613. [Epub ahead of print]. 10.1634/theoncologist.2018-0613 PMC6853097 30936377

[26] Simmons C, McMillan DC, Tuck S, Graham C, McKeown A, Bennett M, et al. How Long Have I Got?—a prospective cohort study comparing validated prognostic factors for use in patients with advanced cancer. Oncologist. 2019. 10.1634/theoncologist.2018-0474. 10.1634/theoncologist.2018-0474 PMC6738290 30975922

[27] Farinholt P, Park M, Guo Y, Bruera E, Hui D (2018). A comparison of the accuracy of clinician prediction of survival versus the palliative prognostic index. J Pain Symptom Manag.

[28] Tavares T, Oliveira M, Gonçalves J, Trocado V, Perpétuo J, Azevedo A, Machado F, Barreto V, Rocha C (2018). Predicting prognosis in patients with advanced cancer: a prospective study. Palliat Med.

[29] Malhotra K, Fenton JJ, Duberstein PR, Epstein RM, Xing G, Tancredi DJ, et al. Prognostic accuracy of patients, caregivers, and oncologists in advanced cancer. Cancer. 2019. 10.1002/cncr.32127. 10.1002/cncr.32127 31034597

[30] Perez-Cruz PE, dos Santos R, Silva TB, Crovador CS, Nascimento MSA, Hall S (2014). Longitudinal temporal and probabilistic prediction of survival in a cohort of patients with advanced cancer. J Pain Symptom Manag.

[31] Brier GW (1950). Verification of forecasts expressed in terms of probability. Mon Weather Rev.

[32] Kee F, Owen T, Leathem R (2007). Offering a prognosis in lung cancer: when is a team of experts an expert team?. J Epidemiol Community Health.

[CR33] White N, Harries P, Harris AJL, Vickerstaff V, Lodge P, McGowan C (2018). How do palliative care doctors recognise imminently dying patients? A judgement analysis. BMJ Open.

[34] Gwilliam B, Keeley V, Todd C, Gittins M, Roberts C, Kelly L, Barclay S, Stone PC (2011). Development of prognosis in palliative care study (PiPS) predictor models to improve prognostication in advanced cancer: prospective cohort study. BMJ..

[35] Gwilliam B, Keeley V, Todd C, Roberts C, Gittins M, Kelly L, Barclay S, Stone P (2013). Prognosticating in patients with advanced cancer—observational study comparing the accuracy of clinicians’ and patients’ estimates of survival. Ann Oncol.

[36] Ericsson KA, Krampe RT, Tesch-Römer C (1993). The role of deliberate practice in the acquisition of expert performance. Psychol Rev.

[37] Ericsson K (2015). Acquisition and maintenance of medical expertise: a perspective from the expert-performance approach with deliberate practice. Acad Med.

[38] White N, Kupeli N, Vickerstaff V, Stone P (2017). How accurate is the ‘Surprise Question’ at identifying patients at the end of life? A systematic review and meta-analysis. BMC Med.

[39] Downar J, Goldman R, Pinto R, Englesakis M, Adhikari NKJ (2017). The “surprise question” for predicting death in seriously ill patients: a systematic review and meta-analysis. CMAJ..

[40] Fromme EK, Smith MD, Bascom PB, Kenworthy-Heinige T, Lyons KS, Tolle SW (2010). Incorporating routine survival prediction in a U.S. hospital-based palliative care service. J Palliat Med.

[41] Zibelman M, Xiang Q, Muchka S, Nickoloff S, Marks S (2014). Assessing prognostic documentation and accuracy among palliative care clinicians. J Palliat Med.

[42] Gripp S, Moeller S, Bölke E, Schmitt G, Matuschek C, Asgari S, Asgharzadeh F, Roth S, Budach W, Franz M, Willers R (2007). Survival prediction in terminally ill cancer patients by clinical estimates, laboratory tests, and self-rated anxiety and depression. J Clin Oncol.

[43] Mackillop WJ, Quirt CF (1997). Measuring the accuracy of prognostic judgments in oncology. J Clin Epidemiol.

[44] Oxenham D, Cornbleet MA (1998). Accuracy of prediction of survival by different professional groups in a hospice. Palliat Med.

[45] Domeisen Benedetti F, Ostgathe C, Clark J, Costantini M, Daud ML, Grossenbacher-Gschwend B, Latten R, Lindqvist O, Peternelj A, Schuler S, Tal K, van der Heide A, Eychmüller S, OPCARE9 (2013). International palliative care experts’ view on phenomena indicating the last hours and days of life. Support Care Cancer.

[46] Taylor P, Dowding D, Johnson M (2017). Clinical decision making in the recognition of dying: a qualitative interview study. BMC Palliat Care..

[47] Stewart Thomas R. (1988). Chapter 2 Judgment Analysis: Procedures. Advances in Psychology.

[48] White N, Oostendorp LJM, Tomlinson C, Yardley S, Ricciardi F, Gökalp H, Minton O, Boland JW, Clark B, Harries P and Stone P. Online training improves medical students’ ability to recognise when a person is dying: the ORaClES randomised controlled trial. Palliat Med in press*.* 10.1177/0269216319880767 PMC6952943 31722611

[49] Eychmüller S, Benedetti FD, Latten R, Tal K, Walker J, Costantini M (2013). ‘Diagnosing dying’ in cancer patients - a systematic literature review. Eur J Palliat Care.

[50] Bruera S, Chisholm G, Dos Santos R, Crovador C, Bruera E, Hui D (2014). Variations in vital signs in the last days of life in patients with advanced cancer. J Pain Symptom Manag.

[51] Hui D, dos Santos R, Chisholm G, Bansal S, Silva TB, Kilgore K, Crovador CS, Yu X, Swartz MD, Perez-Cruz PE, Leite Rde A, Nascimento MS, Reddy S, Seriaco F, Yennu S, Paiva CE, Dev R, Hall S, Fajardo J, Bruera E (2014). Clinical signs of impending death in cancer patients. Oncologist..

[52] Hui D, Dos Santos R, Chisholm G, Bansal S, Souza Crovador C, Bruera E (2015). Bedside clinical signs associated with impending death in patients with advanced cancer: preliminary findings of a prospective, longitudinal cohort study. Cancer..

[53] Hui D, Hess K, Santos R, Chisholm G, Bruera E (2015). A diagnostic model for impending death in cancer patients: preliminary report. Cancer..

[54] Karnofsky D, Burchenal J. The clinical evaluation of chemotherapeutic agents in cancer. In: MacLeod C, editor. Evaluation of chemotherapeutic agents. New York: Columbia University Press. p. 191–205.

[55] Abernethy AP, Shelby-James T, Fazekas BS, Woods D, Currow DC (2005). The Australia-modified Karnofsky Performance Status (AKPS) scale: a revised scale for contemporary palliative care clinical practice. BMC Palliat Care.

[56] Anderson F, Downing GM, Hill J, Casorso L, Lerch N (1996). Palliative performance scale (PPS): a new tool. J Palliat Care.

[57] Oken MM, Creech RH, Tormey DC, Horton J, Davis TE, McFadden ET, Carbone PP (1982). Toxicity and response criteria of the Eastern Cooperative Oncology Group. Am J Clin Oncol.

[58] Downing Michael, Lau Francis, Lesperance Mary, Karlson Nicholas, Shaw Jack, Kuziemsky Craig, Bernard Steve, Hanson Laura, Olajide Lola, Head Barbara, Ritchie Christine, Harrold Joan, Casareti David (2007). Meta-analysis of Survival Prediction with Palliative Performance Scale. Journal of Palliative Care.

[59] Suh S-Y, LeBlanc TW, Shelby RA, Samsa GP, Abernethy AP (2011). Longitudinal patient-reported performance status assessment in the cancer clinic is feasible and prognostic. JOP..

[60] Albain KS, Crowley JJ, LeBlanc M, Livingston RB (1991). Survival determinants in extensive-stage non-small-cell lung cancer: the Southwest Oncology Group experience. J Clin Oncol.

[61] Pirovano M, Maltoni M, Nanni O, Marinari M, Indelli M, Zaninetta G, Petrella V, Barni S, Zecca E, Scarpi E, Labianca R, Amadori D, Luporini G (1999). A new palliative prognostic score: a first step for the staging of terminally ill cancer patients. Italian Multicenter and Study Group on Palliative Care. J Pain Symptom Manag.

[62] Scarpi E, Maltoni M, Miceli R, Mariani L, Caraceni A, Amadori D, Nanni O (2011). Survival prediction for terminally ill cancer patients: revision of the palliative prognostic score with incorporation of delirium. Oncologist..

[63] Morita T, Tsunoda J, Inoue S, Chihara S (1999). The palliative prognostic index: a scoring system for survival prediction of terminally ill cancer patients. Support Care Cancer.

[64] Forrest LM, McMillan DC, McArdle CS, Angerson WJ, Dunlop DJ (2003). Evaluation of cumulative prognostic scores based on the systemic inflammatory response in patients with inoperable non-small-cell lung cancer. Br J Cancer.

[65] Geissbühler P, Mermillod B, Rapin CH (2000). Elevated serum vitamin B12 levels associated with CRP as a predictive factor of mortality in palliative care cancer patients: a prospective study over five years. J Pain Symptom Manag.

[66] Singh S, Cortez D, Maynard D, Cleary JF, DuBenske L, Campbell TC (2017). Characterizing the nature of scan results discussions: insights into why patients misunderstand their prognosis. J Oncol Pract.

[67] Hak T, Koëter G, van der Wal G, The AM (2001). Collusion in doctor-patient communication about imminent death: an ethnographic study. West J Med.

[CR68] Pino M, Parry R (2019). How and when do patients request life-expectancy estimates? Evidence from hospice medical consultations and insights for practice. Patient Educ Couns.

[69] Pino M, Parry R, Land V, Faull C, Feathers L, Seymour J (2016). Engaging terminally ill patients in end of life talk: how experienced palliative medicine doctors navigate the dilemma of promoting discussions about dying. PLoS One.

[70] Paladino Joanna, Lakin Joshua R., Sanders Justin J. (2019). Communication Strategies for Sharing Prognostic Information With Patients. JAMA.

[71] Wright AA, Zhang B, Ray A, Mack JW, Trice E, Balboni T, Mitchell SL, Jackson VA, Block SD, Maciejewski PK, Prigerson HG (2008). Associations between end-of-life discussions, patient mental health, medical care near death, and caregiver bereavement adjustment. JAMA..

[72] Jackson VA, Jacobsen J, Greer JA, Pirl WF, Temel JS, Back AL (2013). The cultivation of prognostic awareness through the provision of early palliative care in the ambulatory setting: a communication guide. J Palliat Med.

[73] Zimmermann C, Swami N, Krzyzanowska M, Leighl N, Rydall A, Rodin G, Tannock I, Hannon B (2016). Perceptions of palliative care among patients with advanced cancer and their caregivers. CMAJ..

[74] Koedoot CG, Oort FJ, de Haan RJ, Bakker PJM, de Graeff A, de Haes JCJM (2004). The content and amount of information given by medical oncologists when telling patients with advanced cancer what their treatment options are: palliative chemotherapy and watchful-waiting. Eur J Cancer.

[75] Clayton JM, Hancock K, Parker S, Butow PN, Walder S, Carrick S, Currow D, Ghersi D, Glare P, Hagerty R, Olver IN, Tattersall MH (2008). Sustaining hope when communicating with terminally ill patients and their families: a systematic review. Psychooncology..

[76] Witkamp FE, van Zuylen L, Vergouwe Y, Rijt, CCD, van der Heide A. Concordance between experiences of bereaved relatives, physicians, and nurses with hospital end-of-life care: everyone has their “Own Truth”. International Journal of Palliative Care. 2015 (Article ID 623890).

[77] Parker SM, Clayton JM, Hancock K, Walder S, Butow PN, Carrick S, Currow D, Ghersi D, Glare P, Hagerty R, Tattersall MH (2007). A systematic review of prognostic/end-of-life communication with adults in the advanced stages of a life-limiting illness: patient/caregiver preferences for the content, style, and timing of information. J Pain Symptom Manag.

[78] Kirk P, Kirk I, Kristjanson LJ (2004). What do patients receiving palliative care for cancer and their families want to be told? A Canadian and Australian qualitative study. BMJ..

[79] Washington Karla T., Craig Kevin W., Parker Oliver Debra, Ruggeri Jeffrey S., Brunk Samantha R., Goldstein Andrea K., Demiris George (2019). Family caregivers’ perspectives on communication with cancer care providers. Journal of Psychosocial Oncology.

[80] Travers A, Taylor V (2016). What are the barriers to initiating end-of-life conversations with patients in the last year of life?. Int J Palliat Nurs.

[81] Bernacki RE, Block SD, American College of Physicians High Value Care Task F (2014). Communication about serious illness care goals: a review and synthesis of best practices. JAMA Intern Med.

[82] Clayton JM, Hancock KM, Butow PN, Tattersall MHN, Currow DC, Australian and New Zealand Expert Advisory Group (2007). Clinical practice guidelines for communicating prognosis and end-of-life issues with adults in the advanced stages of a life-limiting illness, and their caregivers. Med J Aust.

[83] National Institute of Health and Care Excellence. Care of dying adults in the last days of life [NG31]. NICE; 2015. 26741019

[CR84] Royal College of Physicians. Talking about dying: how to begin honest conversations about what lies ahead: Royal College of Physicians; 2018. Evidence-based report providing clinicians clear information about barriers, common myths, recommendations, and further resources to help discussions with patients about their end-of-life care.

[85] Thorne S, Hislop TG, Kuo M, Armstrong E-A (2006). Hope and probability: patient perspectives of the meaning of numerical information in cancer communication. Qual Health Res.

[86] Henselmans I, Smets EMA, Han PKJ, de Haes HCJC, Laarhoven HWM (2017). How long do I have? Observational study on communication about life expectancy with advanced cancer patients. Patient Educ Couns.

[87] Anderson R, Stone P, Low J, Bloch S. P01–098 Discussing prognosis and managing uncertainty in end-of-life conversations with family members: a conversation analytic study. European Association for Palliative Care; 2019. Berlin, Germany: Palliative Medicine.

[88] Bernacki R, Paladino J, Neville BA, Hutchings M, Kavanagh J, Geerse OP, Lakin J, Sanders JJ, Miller K, Lipsitz S, Gawande AA, Block SD (2019). Effect of the serious illness care program in outpatient oncology: a cluster randomized clinical trial. JAMA Intern Med.

[89] Epstein RM, Duberstein PR, Fenton JJ, Fiscella K, Hoerger M, Tancredi DJ, Xing G, Gramling R, Mohile S, Franks P, Kaesberg P, Plumb S, Cipri CS, Street RL Jr, Shields CG, Back AL, Butow P, Walczak A, Tattersall M, Venuti A, Sullivan P, Robinson M, Hoh B, Lewis L, Kravitz RL (2017). Effect of a patient-centered communication intervention on oncologist-patient communication, quality of life, and health care utilization in advanced cancer: the VOICE randomized clinical trial. JAMA Oncol..

[CR90] Paladino Joanna, Bernacki Rachelle, Neville Bridget A., Kavanagh Jane, Miranda Stephen P., Palmor Marissa, Lakin Joshua, Desai Meghna, Lamas Daniela, Sanders Justin J., Gass Jonathon, Henrich Natalie, Lipsitz Stuart, Fromme Erik, Gawande Atul A., Block Susan D. (2019). Evaluating an Intervention to Improve Communication Between Oncology Clinicians and Patients With Life-Limiting Cancer. JAMA Oncology.

[91] Moore PM, Rivera S, Bravo-Soto GA, Olivares C, Lawrie TA (2018). Communication skills training for healthcare professionals working with people who have cancer. Cochrane Database Syst Rev.

[92] Real Talk. Video-based communication training. [Internet] [updated 2019; cited 2019 Sept 23] Available from: https://www.realtalktraining.co.uk/

[93] O'Brien R, Goldberg SE, Pilnick A, Beeke S, Schneider J, Sartain K, Thomson L, Murray M, Baxendale B, Harwood RH (2018). The VOICE study - a before and after study of a dementia communication skills training course. PLoS One.

[94] Jenkins L, Reuber M (2014). A conversation analytic intervention to help neurologists identify diagnostically relevant linguistic features in seizure patients’ talk. Res Lang Soc Interact.

[CR95] Derry HM, Epstein AS, Lichtenthal WG, Prigerson HG (2019). Emotions in the room: common emotional reactions to discussions of poor prognosis and tools to address them. Expert Rev Anticancer Ther.

[96] Piggott KL, Patel A, Wong A, Martin L, Patel A, Patel M, Liu Y, Dhesy-Thind S, You JJ (2019). Breaking silence: a survey of barriers to goals of care discussions from the perspective of oncology practitioners. BMC Cancer.

[97] Kaasa S, Loge JH, Aapro M, Albreht T, Anderson R, Bruera E, Brunelli C, Caraceni A, Cervantes A, Currow DC, Deliens L, Fallon M, Gómez-Batiste X, Grotmol KS, Hannon B, Haugen DF, Higginson IJ, Hjermstad MJ, Hui D, Jordan K, Kurita GP, Larkin PJ, Miccinesi G, Nauck F, Pribakovic R, Rodin G, Sjøgren P, Stone P, Zimmermann C, Lundeby T (2018). Integration of oncology and palliative care: a Lancet Oncology Commission. Lancet Oncol.

[98] Brighton LJ, Selman LE, Bristowe K, Edwards B, Koffman J, Evans CJ (2019). Emotional labour in palliative and end-of-life care communication: a qualitative study with generalist palliative care providers. Patient Educ Couns.

